# Lecithin Inclusion by α-Cyclodextrin Activates SREBP2 Signaling in the Gut and Ameliorates Postprandial Hyperglycemia

**DOI:** 10.3390/ijms221910796

**Published:** 2021-10-06

**Authors:** Eunyoung Lee, Xilin Zhang, Tomoe Noda, Junki Miyamoto, Ikuo Kimura, Tomoaki Tanaka, Kenichi Sakurai, Ryo Hatano, Takashi Miki

**Affiliations:** 1 Department of Medical Physiology, Graduate School of Medicine, Chiba University, Chiba 260-8670, Japan; eylee@restaff.chiba-u.jp (E.L.); x.y.z.hang@chiba-u.jp (X.Z.); tomoenoda3915@chiba-u.jp (T.N.); hatanori@chiba-u.jp (R.H.); 2 Department of Applied Biological Science, Graduate School of Agriculture, Tokyo University of Agriculture and Technology, Fuchu 183-8509, Japan; m-junki@go.tuat.ac.jp; 3 Laboratory of Molecular Neurobiology, Graduate School of Biostudies, Kyoto University, Sakyo-ku, Kyoto 606-8501, Japan; kimura.ikuo.7x@kyoto-u.ac.jp; 4 Department of Molecular Diagnosis, Graduate School of Medicine, Chiba University, Chiba 260-8670, Japan; tomoaki@restaff.chiba-u.jp; 5 Center for Preventive Medical Sciences, Chiba University, Chiba 263-8522, Japan; sakuraik@faculty.chiba-u.jp

**Keywords:** α-cyclodextrin, hyperglycemia, GLP-1, lecithin, inclusion, SREBP2

## Abstract

Background: α-cyclodextrin (α-CD) is one of the dietary fibers that may have a beneficial effect on cholesterol and/or glucose metabolism, but its efficacy and mode of action remain unclear. Methods: In the present study, we examined the anti-hyperglycemic effect of α-CD after oral loading of glucose and liquid meal in mice. Results: Administration of 2 g/kg α-CD suppressed hyperglycemia after glucose loading, which was associated with increased glucagon-like peptide 1 (GLP-1) secretion and enhanced hepatic glucose sequestration. By contrast, 1 g/kg α-CD similarly suppressed hyperglycemia, but without increasing secretions of GLP-1 and insulin. Furthermore, oral α-CD administration disrupts lipid micelle formation through its inclusion of lecithin in the gut luminal fluid. Importantly, prior inclusion of α-CD with lecithin in vitro nullified the anti-hyperglycemic effect of α-CD in vivo, which was associated with increased intestinal mRNA expressions of SREBP2-target genes *(Ldlr*, *Hmgcr*, *Pcsk9*, and *Srebp2*). Conclusions: α-CD elicits its anti-hyperglycemic effect after glucose loading by inducing lecithin inclusion in the gut lumen and activating SREBP2, which is known to induce cholecystokinin secretion to suppress hepatic glucose production via a gut/brain/liver axis.

## 1. Introduction

The number of patients with metabolic disease, such as diabetes mellitus and obesity has been increasing worldwide, and the medical cost for treatment has become a social burden [1]. To cope with these issues, better understanding of the pathophysiology and establishment of innovative therapy is critical. In addition, a preventive healthcare approach is especially beneficial to those at risk of the diseases. While proper exercise and diet is known to be effective in preventing metabolic diseases [2], various dietary supplements can reinforce life style interventions. As one example of such an approach, in 1991, the Japanese government started a health promoting policy in which the ‘Consumer Affairs Agency’ certifies a food beneficial to health as a ‘Food for Specified Health Uses (FOSHU)’ [3]. The targeted diseases of FOSHU cover a wide range of physiological conditions, such as hypertension, dyslipidemia, obesity, and hyperglycemia. Among these, foods claimed to ameliorate postprandial hyperglycemia have the largest market share among all approved FOSHU. Active ingredients of this category include L-arabinose, salacia (*Salacia chinensis*) root extract containing neokotalanol, guava (*Psidium guajava*) leaf extract containing polyphenol, and wheat albumin; resistant dextrin being the most widely used. Resistant dextrin is a water-soluble dietary fiber and is added to FOSHU to alleviate postprandial hyperglycemia. Foods containing resistant dextrin account for >30% of FOSHU in Japan. Resistant dextrin is poorly hydrolysed by α-glucosidases contained in digestive juice [4]. As a result, over 90% of orally indigested resistant dextrin reaches the large intestine without being hydrolyzed and absorbed by the host. Instead, half is utilized by gut microbiota in the large intestine and the rest is excreted as feces [5]. In Japan, many kinds of beverage containing resistant dextrin have been approved as FOSHU with anti-hyperglycemic action when taken together with meal. However, its mechanism of action has not been fully evaluated.

Interestingly, Wakabayashi et al. have reported that the anti-hyperglycemic effect of resistant dextrin is mediated through its property as an α-glucosidase inhibitor (α-GI) [6]. This might suggest that resistant dextrin elicits an anti-hyperglycemic effect simply by delaying digestion and absorption of carbohydrates. However, recent studies have clarified that soluble dietary fibers including resistant dextrin can be utilized by gut microbiota, and that their metabolites have been shown to play a critical role in regulation of host metabolism through various mechanisms [7]. In accord with this, we recently reported that carbohydrates in the gut lumen can be utilized by gut microbiota to modulate incretin secretion [8,9]. In the present study, we have clarified the effect of dietary fibers on glycemic changes after carbohydrate loading in mice and elucidated the underlying mechanisms.

## 2. Results

### 2.1. Resistant Dextrin Exhibits Modest Effects on Suppression of Hyperglycemia after Co-Administration of Glucose or Liquid Meal in Mice

**Figure 1:**
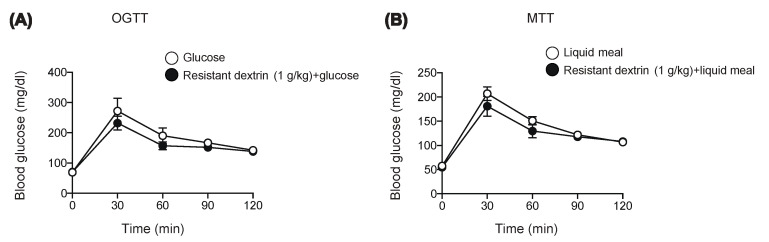
Effect of resistant dextrin on glycemia after glucose or meal loading. Blood glucose levels were measured after glucose (1 g/kg) (**A**) or liquid meal (10 μL/g BW) (**B**) in WT mice. Bars depict mean ± SEM (*n* = 4–5).

We first examined the effect of resistant dextrin on blood glucose after loading monosaccharide glucose (Figure 1A) or liquid meal (Figure 1B) in mice. In accord with the previous reports [6], resistant dextrin did not lower blood glucose levels after oral glucose administration (Figure 1A). Furthermore, 1 g/kg resistant dextrin barely suppressed hyperglycemia after loading of liquid meal containing dextrin as the nutrient source (1 g/kg carbohydrate). We then examined whether another type of dietary fiber might exert a more robust anti-hyperglycemic effect in response to carbohydrate ingestion.

### 2.2. α-CD Administration with Glucose Decreases Blood Glucose via GLP-1 Dependent and Independent Pathways

**Figure 2:**
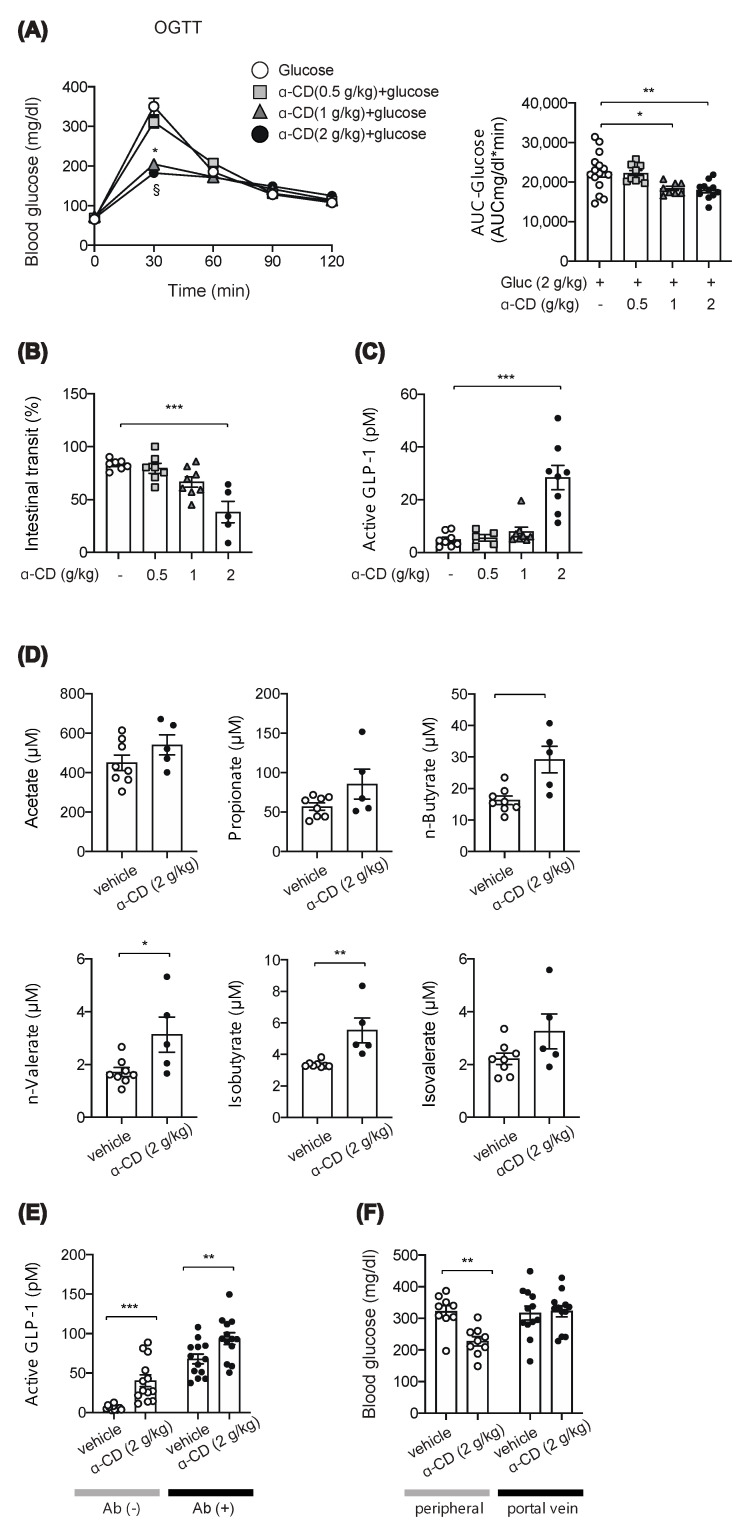
Effect of 2 g/kg α-CD on blood glucose and GLP-1, intestinal transit and SCFA levels. (**A**) Blood glucose was measured after glucose (2 g/kg) loading with or without α-CD (0.5, 1, 2 g/kg) (*n* = 8–15). * *p* < 0.001 (control vs. 1 g/kg α-CD), and § *p* < 0.001(control vs. 2 g/kg α-CD). (**B**) Small intestinal transit in WT mice pre-administered with α-CD (0.5, 1, 2 g/kg) (*n* = 5–7). (**C**) Plasma GLP-1 levels in the portal vein (0.5, 1, 2 g/kg) (*n* = 5–8). (**D**) Plasma SCFA levels in the portal vein at 30 min after 2 g/kg α-CD administration (*n* = 6–8). (**E**) Plasma GLP-1 levels in the portal vein at 30 min after 2 g/kg α-CD administration in the WT mice with [Abs (+)] or without [Abs (−)] Abs-treatment (*n* = 13). (**F**) Blood glucose levels in peripheral and portal vein at 30 min after 2 g/kg α-CD administration (*n* = 9–12). Each data is plotted as a circle. Bars depict mean ± SEM. * *p* < 0.05, ** *p* < 0.01, and *** *p* < 0.001 by one-way ANOVA (**A**–**C**) plus Tukey’s post hoc analysis (**A**–**C**) or by unpaired-Student *t*-test (**D**–**F**).

We focused on α-cyclodextrin (α-CD), which has been reported to ameliorate hyperglycemia after carbohydrate loading [10,11]. However, the mechanism of anti-hyperglycemic effect of α-CD remains unknown; several in vitro studies reported that α-CD inhibits α-amylase activity [12,13], which may account for the glucose lowering effect of α-CD when taken with rice or bread in humans [10,14]. To clarify involvement of its α-GI property, we first evaluated the anti-hyperglycemic effect of α-CD by co-administration with monosaccharide glucose. Unexpectedly, the rise in glycemia in response to glucose was markedly suppressed by 2 g/kg and 1 g/kg α-CD (*p* < 0.001 vs. control for both 2 g/kg and 1 g/kg α-CD) (Figure 2A), suggesting that α-CD elicits an anti-hyperglycemic effect through a mechanism independent from its α-GI activity.

To distinguish the mechanism of α-CD in suppressing hyperglycemia, we measured intestinal transit; 2 g/kg α-CD administration significantly inhibited intestinal transit, while 1 g/kg or 0.5 g/kg α-CD administration did not do so (Figure 2B). As the gut hormone GLP-1 is known to delay gastric emptying [15], we measured GLP-1 secretion in the portal vein at 30 min after α-CD administration. Interestingly, 2 g/kg α-CD administration markedly induced GLP-1 secretion, while 1 g/kg or 0.5 g/kg α-CD administration did not stimulate GLP-1 secretion (Figure 2C).

Chronic α-CD supplementation has been shown to increase the concentration of short chain fatty acids (SCFAs) [16], which trigger GLP-1 secretion [17]. In addition, we previously reported that a single administration of maltose plus miglitol (an α-glycosidase inhibitor) resulted in the increase in SCFA levels in the portal vein [9]. Therefore, we measured SCFA levels in the portal vein at 30 min after an acute, single administration of 2 g/kg α-CD; SCFA levels were significantly increased by 2 g/kg α-CD (n-Butyrate (*p* < 0.01), *n*-Valerate (*p* < 0.05), and Isobutyrate (*p* < 0.01) (Figure 2D), indicating that a single administration of α-CD acutely increases the SCFAs produced directly or indirectly from α-CD by gut microbiota, which may stimulate GLP-1 secretion. Nevertheless, we also think that GLP-1 secretion by 2 g/kg α-CD is triggered through a SCFA-independent mechanism, as well as through a SCFA-dependent mechanism; α-CD-induced GLP-1 secretion was significantly increased (*p* < 0.01 vs. vehicle) even in ‘dysbiotic’ mice produced by multiple antibiotic treatment for 4 weeks, in which SCFA production was markedly reduced [9] (Figure 2E). However, as we did not examine the effect of α-CD on the SCFA levels, it remains uncertain whether or not α-CD is utilized to from SCFAs in these mice.

We also evaluated the physiological mechanism for the marked prevention of hyperglycemia by α-CD. It might be induced extra-hepatically (i.e., by decreased glucose absorption from the gut due to suppressed gastric emptying) or hepatically (i.e., by increased glucose sequestration either through suppressed glucose production or increased glucose uptake in the liver). To determine which, we measured blood glucose levels in peripheral and portal vein after administration of glucose with or without 2 g/kg α-CD. Intriguingly, α-CD significantly lowered glucose levels in the peripheral vein (*p* < 0.01), but not in the portal vein (Figure 2F), indicating that α-CD prevents hyperglycemia through increasing hepatic glucose sequestration and not by suppressing glucose absorption due to reduced intestinal motility.

### 2.3. α-CD Suppresses Meal-Induced Hyperglycemia via an Insulin-Independent Pathway

**Figure 3:**
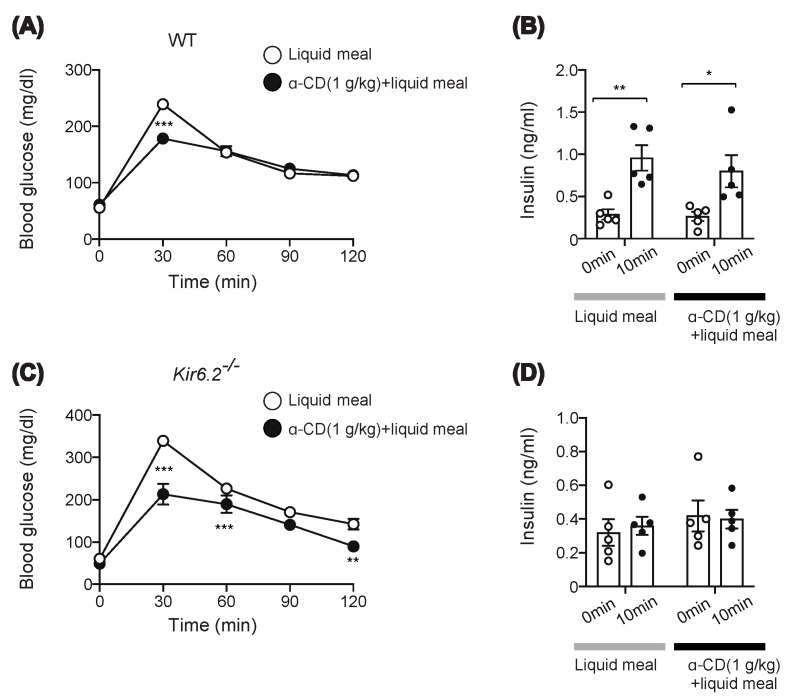
Effect of 1 g/kg α-CD on blood glucose and plasma insulin levels during MTT. (**A**,**C**) Blood glucose levels during MTT using liquid meal (10 μL/g) supplemented with or without α-CD (1 g/kg) in WT (**A**) (*n* = 8) and *Kir6.2*^−/−^ mice (**C**) (*n* = 9). (**B**,**D**) Plasma insulin levels before and at 10 min during MTT in WT (**B**) (*n* = 5) and *Kir6.2*^−/−^ mice (**D**) (*n* = 5). Bars depict mean ± SEM. Each data is plotted as a circle. * *p* <0.05, ** *p* < 0.01, and *** *p* < 0.001 by unpaired-Student *t*-test.

Intriguingly, 1 g/kg α-CD administration had an apparent anti-hyperglycemic effect, but without triggering GLP-1 secretion or delaying intestinal transit, suggesting that 1 g/kg α-CD might ameliorate hyperglycemia through a GLP-1-independent mechanism. Since we did not measure the rise in SCFAs by 1 g/kg α-CD, the involvement of SCFAs in the anti-hyperglycemic effect of 1 g/kg α-CD remains to be clarified. We performed a meal tolerance test (MTT) rather than an oral glucose tolerance test (OGTT) to account for more physiological feeding conditions, and measured blood glucose and insulin levels. Consistent with the OGTT results (Figure 2A), α-CD suppressed meal-induced hyperglycemia in wild-type (WT) mice (Figure 3A). Unexpectedly, 1 g/kg α-CD did not potentiate insulin secretion during MTT (Figure 3B). Since an anti-hyperglycemic effect was induced by α-CD without potentiating insulin secretion in WT mice, we expected that a similar effect would be induced in K_ATP_ channel-deficient *Kir6.2*^−/−^ mice, in which the insulin secretory response to meal was severely impaired [18]. As expected, the rise in blood glucose in response to meal was significantly suppressed by co-administration of 1 g/kg α-CD (*p* < 0.001 vs. control in *Kir6.2*^−/−^ mice) (Figure 3C), even though insulin secretion was severely impaired in *Kir6.2*^−/−^ mice (Figure 3D). Taken together, these results suggest that the blood glucose-lowering effect of 1 g/kg α-CD is exerted through an insulin-independent mechanism.

### 2.4. Preinclusion of α-CD with Lecithin Abolishes Glucose-Lowering Effect of α-CD

**Figure 4:**
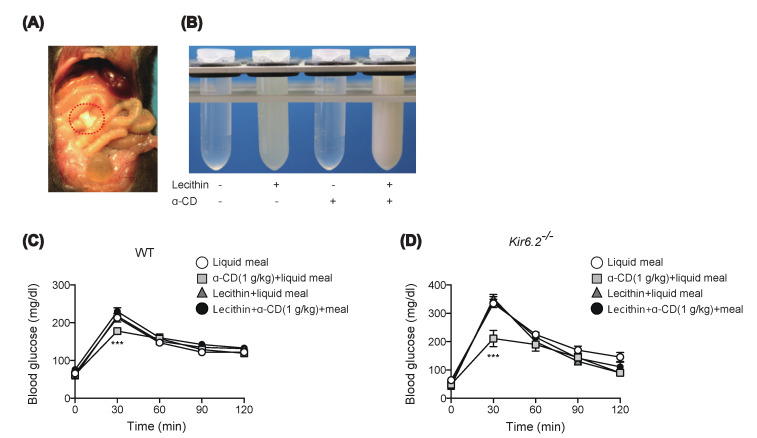
Effect of preinclusion of 1 g/kg α-CD with lecithin during MTT. (**A**) Appearance of small intestinal content (circled by a red, dotted line) at 30 min after α-CD administration. A small incision was made in the small intestine to induce the outflow the intestinal fluid. (**B**) Macroscopic appearance of test samples. Mixing α-CD and lecithin induced liquid clouding, resembling that observed in vivo (**A**). (**C**,**D**) Effect of preinclusion of α-CD with lecithin during MTT in WT (**C**) (*n* = 9) and *Kir6.2*^−/−^ mice (**D**) (*n* = 5). Bars depict mean ± SEM. *** *p* < 0.001 by unpaired-Student *t*-test.

We then investigated the mechanism of the anti-hyperglycemic effect of 1 g/kg α-CD. On sampling the blood from the portal vein, we noticed that intestinal content looked cloudy after α-CD administration plus glucose (Figure 4A). As inclusion of lecithin by α-CD has been reported to inhibit formation of bile salt micelles, which leads to inhibition of cholesterol absorption by the host [19], inclusion of lecithin by α-CD in intestinal fluid might well lead to the formation of a cloudy substance consisting of α-CD and lecithin; we therefore mixed α-CD and lecithin in aqueous solution and observed its liquid property in test tubes. While α-CD is water-soluble, lecithin suspension appears slightly cloudy in water. In accord with a previous report [19], when α-CD solution was added to lecithin suspension, the liquid became cloudy (Figure 4B), with a similar appearance to that occurring in the gut lumen after α-CD administration in vivo (Figure 4A). Should orally administered α-CD pull out lecithin from the intraluminal fat micelles, it could result in destruction of the fat micelles, which are essential for cholesterol supply to the gut epithelium. Thus, the anti-hyperglycemic effect of α-CD may well be exerted through destruction of fat micelles via lecithin depletion. To test this, we administered WT and *Kir6.2*^−/−^ mice with α-CD pre-mixed with lecithin (lecithin: α-CD = 1:3 molecular ratio). Pre-inclusion of α-CD with lecithin completely abolished the anti-hyperglycemic effect of α-CD in both genotypes after administration of 1 g/kg α-CD plus 0.26 g/kg lecithin (Figure 4C,D), indicating that the inclusion property of α-CD is necessary to elicit its anti-hyperglycemic effect. However, it remains unknown whether the orally administered α-CD acts on intraluminal fat micelles, which must be clarified in future study.

### 2.5. α-CD Administration Activates Intestinal SREBP2 Signaling

**Figure 5:**
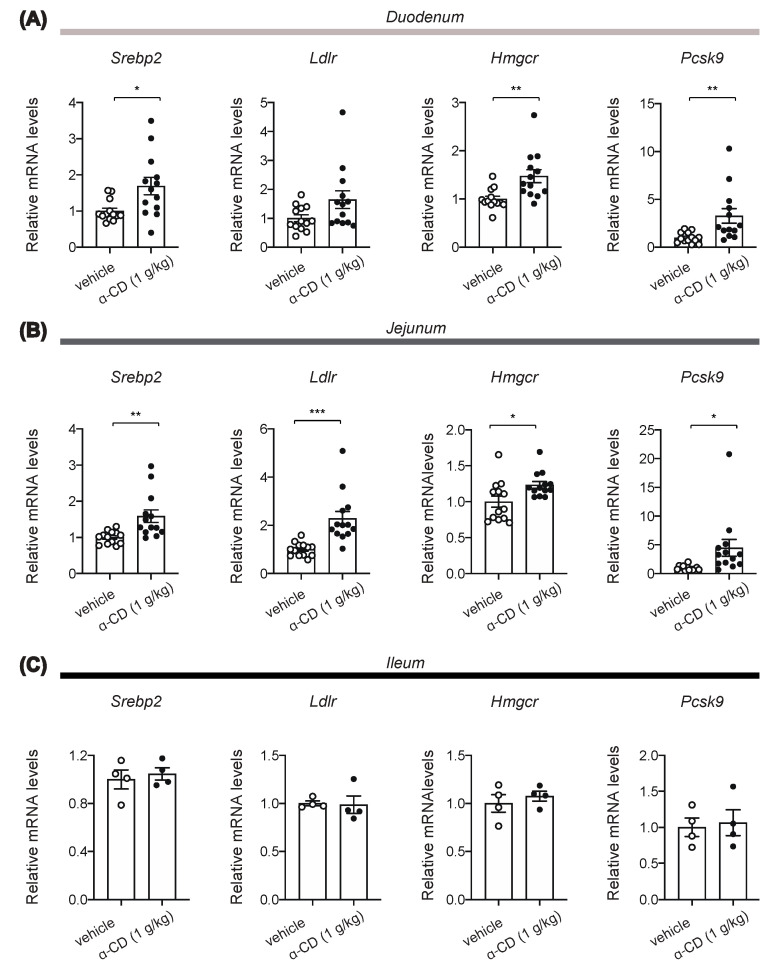
Induction of SREBP2 target gene expressions in small intestine by α-CD. (**A**–**C**) Effects of α-CD on mRNA expression of SREBP2 target genes (*Srebp2*, *Ldlr*, *Hmgcr*, and *Pcsk9*) in duodenum (**A**) (*n* = 13–14), jejunum (**B**) (*n* = 13–14), and ileum (**C**) (*n* = 4). Bars depict mean ± SEM. Each data is plotted as a circle. * *p* < 0.05, ** *p* < 0.01, and *** *p* < 0.001 by unpaired-Student *t*-test.

Extracellular cholesterol concentration is sensed by absorptive epithelial cells and hepatocytes through SREBP2 signaling [20]. In addition, it has been reported that activation of SREBP2 in the duodenum directly induces cholecystokinin (CCK) secretion from enteroendocrine I-cells [21], and that locally increased CCK in the gut suppresses hepatic glucose production via a gut-brain-liver axis [22]. Indeed, our experiments reveal that 1 g/kg α-CD elicits its anti-hyperglycemic effect without potentiating insulin secretion. Thus, blockade of the cholesterol supply to gut epithelium by α-CD may well activate SREBP2 in intestine, thus stimulating CCK secretion that suppresses hepatic glucose production. We accordingly assessed expression of SREBP2 target genes at 30 min after α-CD administration. Intriguingly, mRNA expressions of SREBP2 target genes such as *Ldlr*, *Hmgcr*, *Pcsk9*, and *Srebp2* (autocrine induction) were significantly increased by α-CD administration in duodenum and jejunum (Figure 5A, B) but not in ileum (Figure 5C).

## 3. Discussion

Although ingestion of FOSHU containing resistant dextrin together with meal is generally considered to diminish postprandial hyperglycemia in Japan, the results of our protocol in mice do not support its beneficial effect on glycemia. We then examined the effects of another type of dietary fiber, α-CD, on anti-hyperglycemia in response to carbohydrate ingestion. α-CD is a cyclic oligosaccharide consisting of six glucose residues linked with α 1,4-glycosidic bonds [23], which is poorly hydrolyzed by pancreatic enzymes and is barely absorbed in the small intestine. For this reason, administered α-CD reaches the large intestine mostly intact, where it is fermented by gut microbiota, leading to increased SCFA production when ingested chronically [16]. In addition, several in vitro studies reported that α-CD inhibits α-amylase activity, presumably due to the competitive binding of α-CD to its active site [12,13]. In accord with this, α-CD has been reported to have a blood glucose-lowering effect when taken with white rice or white bread in humans [10,14]. However, in contrast to these reports, Bessell et al. recently reported that chronic α-CD intake for 6 months did not result in any changes of blood glucose or insulin levels [24]. Furthermore, Gentilcore et al. reported that α-CD did not reduce the glycemic response to 100 g sucrose load in healthy subjects [25]. Thus, the anti-hyperglycemic effect of α-CD and the contribution of its α-glucosidase activity to the effect remain elusive.

In our present study, we found that α-CD elicits anti-hyperglycemic effect after co-administration with monosaccharide glucose, indicating that it occurs through a mechanism independent of its α-GI activity. We also found that the anti-hyperglycemic effect of α-CD differs depending on the dosages used. 2 g/kg α-CD was shown to induce GLP-1 secretion associated with increase in SCFAs in the portal vein. SCFAs produced by gut microbiota stimulate GLP-1 secretion via its binding to the SCFA receptor GPR43 [17]. Our present study indicates that a single administration of α-CD can acutely increase microbiota-borne SCFAs, which may contribute to an increase in GLP-1 secretion. Our results further suggest that α-CD also elicits GLP-1 secretion through a SCFA-independent mechanism. Notably, 2 g/kg α-CD administration decreased blood glucose levels in the peripheral vein (*p* < 0.01), but not in the portal vein, indicating that α-CD prevents hyperglycemia through increasing hepatic glucose sequestration, but not through suppression of glucose absorption in the gut due to reduced intestinal motility. Taken together, these results suggest that 2 g/kg α-CD induces SCFA production and GLP-1 secretion, which may contribute to its anti-hyperglycemic effect. In addition, while 2 g/kg α-CD suppresses gastric emptying, the anti-hyperglycemic effect of α-CD is likely to be mediated mainly by increased hepatic glucose sequestration.

By contrast, 1 g/kg α-CD administration showed apparent anti-hyperglycemic effect, but without triggering GLP-1 secretion or delaying intestinal transit, suggesting that 1 g/kg α-CD elicits its anti-hyperglycemic effect through a GLP-1-independent mechanism. In addition, 1 g/kg α-CD did not potentiate insulin secretion, yet effectively suppressed hyperglycemia in K_ATP_ channel deficient *Kir6.2*^−/−^ mice, a model of defective insulin secretion in response to meal, as well as in WT mice, indicating that the blood glucose-lowering effect of 1 g/kg α-CD is exerted through an insulin-independent mechanism.

During the course of the study, we found that α-CD forms a cloudy precipitate in the gut when administered orally. α-CD forms inclusions with small sized molecules in its hydrophobic inner cavity. Because of this molecular characteristic, α-CD has been used widely as a pharmaceutical ingredient to improve drug solubility and bioactivity [26]. When α-CD is administered intra-luminally to the gut, inclusion of lecithin by α-CD has been reported to inhibit formation of bile salt micelles, leading to inhibition of cholesterol absorption by the host [19]. In the gut lumen, bile salts and lecithin form micelles with triglycerides and cholesterol, which are critical for absorption of dietary fat. To ascertain whether the anti-hyperglycemia effect of α-CD is exerted through destruction of fat micelles via lecithin depletion, we tested whether administration of α-CD that had been premixed with lecithin during MTT was effective. The complete lack of effect of α-CD premixed with lecithin during MTT established that inclusion of lecithin in the gut by α-CD is essential for eliciting its anti-hyperglycemic effect.

Considering the importance of lipid micelles in the absorption of lipids, including cholesterol, by the gut epithelium, α-CD might well evoke a cholesterol-depleting signal in the gut. The extracellular cholesterol concentration is sensed by absorptive epithelial cells and hepatocytes through SREBP2 signaling [20]. In addition, SREBP2 activation in the gut directly induces CCK secretion from I-cells [22], which are most abundant in duodenum [27,28], suggesting that α-CD could induce CCK secretion from I-cells through activation of SREBP2 signaling. It remains unknown whether SREBP2 activation by α-CD suppresses hepatic glucose production, which should be clarified in future studies.

To date, although several investigations have reported an anti-hyperglycemic effect of α-CD in both clinical and non-clinical studies, the results are inconsistent. Some clinical studies find support for the anti-hyperglycemic effect of α-CD [10,14], while others do not [24,29]. Our current study was performed in mice at a high dose (1 g/kg or 2 g/kg) of α-CD given in an acute, single administration. Although α-CD has been approved to be safe by the FDA (Food and Drug Administration), the agency responsible for the control and safety of food and drugs in the U.S. [30], 25g α-CD was shown to induce mild bloating and diarrhea in healthy subjects in a clinical study [14]. Therefore, clinical efficacy and safety of α-CD should be examined in the future studies. Alternatively, some other molecules mimicking α-CD action might be more effective in reducing the hyperglycemia for subjects at risk of diabetes mellitus.

In conclusion, we found that an acute, single administration of α-CD to mice evokes a potent anti-hyperglycemic effect after glucose loading by inducing inclusion of the substance (including the lecithin) in the gut lumen and possibly by activating SREBP2 signaling in the duodenum and jejunum. Considering that SREBP2 activation has been reported to induce CCK secretion from enteroendocrine I-cells and that CCK inhibits hepatic glucose production via a gut/brain/liver axis, the anti-hyperglycemic effect of α-CD is likely to be mediated by a mechanism involving CCK signaling in the gut. The involvement of SREBP2 signaling in anti-hyperglycemia effect of α-CD needs to be clarified in the future.

## 4. Materials and Methods

### 4.1. Animals

Male wild-type (WT) mice and *Kir6.2*^−/−^ mice [31] with a genetic background of C57BL/6J were used for the study. WT mice were purchased from Japan SLC, Inc. (Shizuoka, Japan). The mice were maintained on normal standard chow diet (CE-2) (12.1% kcal from fat) (CLEA Japan Inc., Tokyo, Japan). All animal studies were approved by the Animal Care and Use Committee of Chiba University (A30-223, A2-095, and A3-052; approved on 20 March 2019, 19 March 2020, and 24 March 2021, respectively).

### 4.2. Oral Glucose Tolerance Test and Meal Tolerance Test

#### 4.2.1. Procedures for Oral Glucose or Meal Tolerance Test

OGTT and MTT were performed by oral gavage of 20% (*w/v*) glucose solution or liquid meal (10 μL/g BW) (Ensure Liquid, Abbot Japan Co., Ltd., Tokyo, Japan) with or without dietary fiber (resistant dextrin, α-CD, or lecithin-premixed α-CD, see below) to mice fasted for 16 h. Blood glucose levels were measured as previously reported [32] at the time points indicated in the figures (Figure 1A,B, Figure 2A, Figure 3A,C, and Figure 4C,D).

#### 4.2.2. Preparation of Resistant Dextrin, α-CD, and Lecithin and Their Use for In Vivo Experiments

D-glucose, resistant dextrin, α-CD, and lecithin from egg were purchased from Nacalai Tesque (Code No. 16805-35) (Kyoto, Japan), Daitobussan Co. (Code No. p000014) (Hiroshima, Japan), Tokyo Chemical Industry Co., Ltd. (Code No. C0776) (Tokyo, Japan), and Fuji Film (Code No. 124-05031) (Osaka, Japan), respectively. For 2 g/kg OGTT, 20% (*w/v*) glucose solution was given via gavage. For OGTT with 2 g/kg glucose plus α-CD (either 0.5, 1, or 2 g/kg), 20% (*w/v*) glucose solution containing each dose of α-CD [5, 10, and 20% (*w/v*), respectively] was prepared and given via gavage. For MTT with premixed α-CD and lecithin, liquid meal containing 10% (*w/v*) α-CD and 26 mg/mL lecithin was prepared and given via gavage.

### 4.3. Preparation of α-CD and Lecithin and Their Use for In Vitro Experiments

For in vitro experiment mixing α-CD and lecithin in test tubes, three types of solution in water (vehicle (water), 10% (*w/v*) α-CD solution, and 26 mg/mL lecithin suspension) were prepared. Two of these (as depicted in Figure 4B) were mixed together at 1:1 volume ratio and their liquid properties were examined macroscopically.

### 4.4. Measurement of Portal GLP-1, SCFAs, and Insulin

Plasma levels of GLP-1, SCFAs, and insulin in the portal vein were measured at 30 min after α-CD administration (for GLP-1 and SCFAs, Figure 2C–E) or at 10 min after glucose administration (for insulin, Figure 3B,D) to mice as previously reported [8,9,33]. Briefly, 2 g/kg α-CD or vehicle (water) was orally administered to overnight-fasted mice. Thirty minutes after ingestion, the mice were anesthetized with isoflurane for 3 min and laparotomized to collect portal vein blood, which was subjected to measurement of blood glucose and plasma GLP-1, SCFAs, and insulin. Plasma GLP-1 [8] and insulin [33] concentrations were measured using commercially available ELISA kits, as previously described. Plasma levels of the SCFAs (Acetate, Propionate, *n*-Butyrate, *n*-Valerate, Isobutyrate, and Isovalerate) in the portal vein were measured as previously described [9].

### 4.5. Intestinal Transit

Mice were fasted for 16 h and given oral gavage of vehicle (water) or α-CD (0.5, 1, 2 g/kg). Thirty min later, 10% glucose solution containing 5% Evans Blue dye (Sigma-Aldrich Co. LLC (St. Louis, MO, USA)) was further administered; small intestinal transit was assessed 10 min later. After euthanasia and dissection of small intestine, the length from the pylorus to the most distal point of migration of the Evans Blue (A) and the length of the small intestine (B) was measured. Gastrointestinal transit was expressed as percentage of A to B.

### 4.6. Quantitative Real-Time PCR

Total RNA was isolated from the gut mucosa (duodenum, proximal jejunum, and terminal ileum) and subjected to qPCR analyses using Fast SYBR^®^ Green Master Mix (Applied Biosystems, Waltham, MA, USA), according to the manufacturer’s protocol, as previously described [34]. After euthanizing the mice, a laparotomy was performed and the gut samples were excised; duodenum, proximal jejunum, and terminal ileum were collected from ~3 cm segments distal from the pylorus, ~3 cm segments distal from the ligament of Treitz, and ~3 cm segments proximal to the cecum, respectively. Gut segments were cut open longitudinally and the lumen was flushed with ice-cooled PBS and gut mucosa was scraped with a steel spatula and immediately stored in RNAlater^TM^ (Thermo Fisher Scientific, Waltham, MA, USA) at 4 °C overnight before subsequent storage at −80 °C until RNA extraction.

The primers used were 5′-acccttcaccaatgactcctatg-3′ and 5′-atgatgactgcagcaaatcgc-3′ for *Tbp*, 5′-caagcacactgattgagat-3′ and 5′-tgggcacgatttaagaagta-3′ for *Srebp2*, 5′-tgcctggatgggaaggagta-3′ and 5′-gcctcgagtcatcccatctg-3′ for *Hmgcr*, 5′-gaggagcagccacatggtat-3′ and 5′-gctcgtcctctgtggtcttc-3′ for *Ldlr*, 5′-ccccatgtggagtacattga-3′ and 5′-gtggaagcgtgtcccatc-3′ for *Pcsk9*.

### 4.7. Statistical Analysis

Results are expressed as means ± SEM. Differences between two groups were assessed using the unpaired two-tailed Student’s *t*-test unless otherwise noted. Data sets involving more than two groups were assessed by One-way ANOVA or Two-way ANOVA (GraphPad Prism software, version 8.0) (GraphPad Software, San Diego, CA, USA). *p* < 0.05 was considered statistically significant.

## Data Availability

Not applicable.

## Abbreviations

α-CD	α-cyclodextrin
GLP-1	glucagon-like peptide 1
FOSHU	Food for Specified Health Uses
α-GI	α-glucosidase inhibitor
SCFAs	short chain fatty acids
MTT	meal tolerance test
OGTT	oral glucose tolerance test
CCK	cholecystokinin
FDA	Food and Drug Administration
